# Intravascular ultrasound imaging of isolated and non aorto-ostial coronary Takayasu arteritis: a case report

**DOI:** 10.1186/s12872-020-01541-x

**Published:** 2020-06-01

**Authors:** Takeshi Shimizu, Akihiko Sato, Keiji Sakamoto, Yoshitane Seino, Mikihiro Kijima, Toshiharu Matsumoto, Yasuchika Takeishi

**Affiliations:** 1 Department of Cardiovascular Medicine, Fukushima Medical University, 1 Hikarigaoka Fukushima, Fukushima, 960-1247 Japan; 2 Department of Cardiology, Hoshi General Hospital, Koriyama, Japan; 3 Department of Diagnostic Pathology, Juntendo University Nerima Hospital, Tokyo, Japan

**Keywords:** Takayasu arteritis, Non aorto-ostial coronary stenosis, Intravascular ultrasound, Pathology, Case report

## Abstract

**Background:**

Isolated coronary Takayasu arteritis is a rare form of ischemic heart disease that typically appears as an aorto-ostial lesion. Although several vascular imaging modalities including ultrasonography, computed tomographic angiography, magnetic resonance angiography or catheter angiography, play crucial roles for diagnosing Takayasu arteritis, the intravascular ultrasound imaging of Takayasu arteritis is not well studied.

**Case presentation:**

A 55-year-old woman who was diagnosed with heterozygous familial hypercholesterolemia underwent coronary angiography due to effort angina, which showed ostial left anterior descending coronary artery (LAD) stenosis. Although directional coronary atherectomy followed by drug-coated balloon was successfully performed, 6 months later restenosis occurred at the ostial LAD, and the ostial left circumflex coronary artery (LCx) progressed significantly. The intravascular ultrasound imaging in these lesions was noteworthy, in which the media was partly unrecognizable and an echo intensity similar to fibrotic intimal thickening traversed from the intima to the adventitia, thereby causing the whole image of the coronary artery to become unclear. Directional coronary atherectomy followed by drug-coated balloon procedures for both LAD and LCx lesions were performed again. Systemic examination of computed tomographic angiography found no other stenotic lesions except for those in the coronary arteries. Five months later, the LAD and LCx lesions progressed diffusely, therefore the coronary artery bypass graft was done. The histopathological findings of specimens of the coronary artery that were obtained during the bypass graft showed excessive fibrous thickening of the intima and adventitia, with granulomatous inflammation in the media, which led to the diagnosis of isolated coronary Takayasu arteritis. Systemic corticosteroid therapy was then started.

**Conclusions:**

We described an extremely rare case of isolated and non aorto-ostial Takayasu arteritis. The characteristic intravascular ultrasound images of diseased coronary arteries may help in the diagnosis of coronary Takayasu arteritis.

## Background

Takayasu arteritis is an idiopathic chronic inflammatory arteritis characterized by stenosis, occlusion or dilatation of the aorta and its major branches, predominantly prevalent among young women [1]. Coronary artery involvement has been reported in approximately 10 to 45% of autopsy cases of Takayasu arteritis [2, 3]. Since over 70% of coronary lesions in Takayasu arteritis are located at the coronary ostium [2, 4], diagnosing Takayasu arteritis is difficult when the lesion is isolated in the coronary artery and not at the aorto-ostium. To make the diagnosis of Takayasu arteritis, both clinical symptoms caused by ischemia of particular organs and characteristic vascular findings of diagnostic images, including ultrasonography, computed tomography (CT) angiography, magnetic resonance angiography or catheter angiography, play crucial roles. However, the intravascular ultrasound (IVUS) imaging of Takayasu arteritis is not well studied. We here present an extremely rare case of isolated and non aorto-ostial Takayasu arteritis that was diagnosed histopathologically. In the current case, IVUS images of the coronary lesion revealed characteristic findings. The characteristic IVUS imaging might help for diagnosing coronary Takayasu arteritis.

## Case presentation

**Fig. 1:**
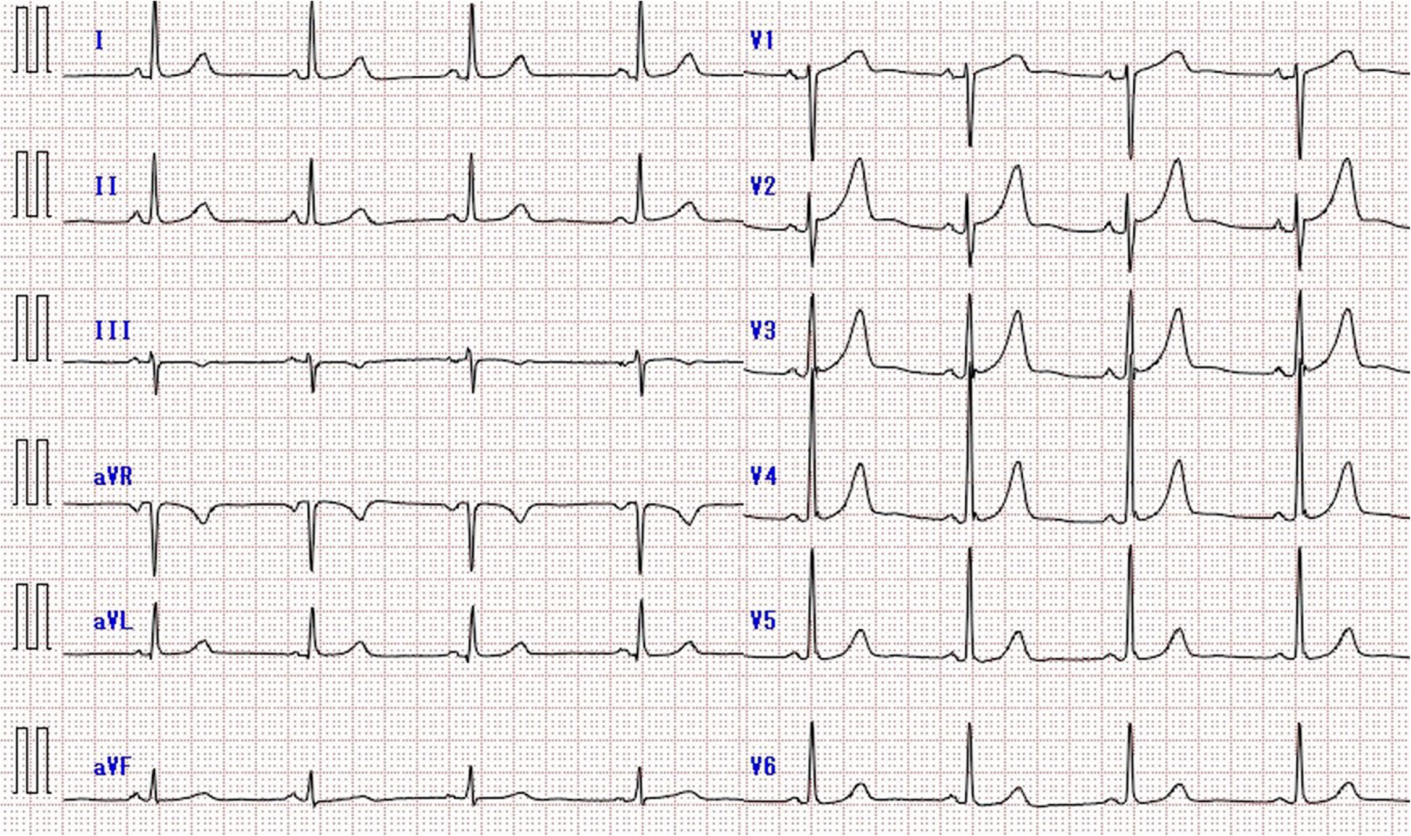
Echocardiogram. Showing regular sinus rhythm and no significant abnormalities

**Fig. 2:**
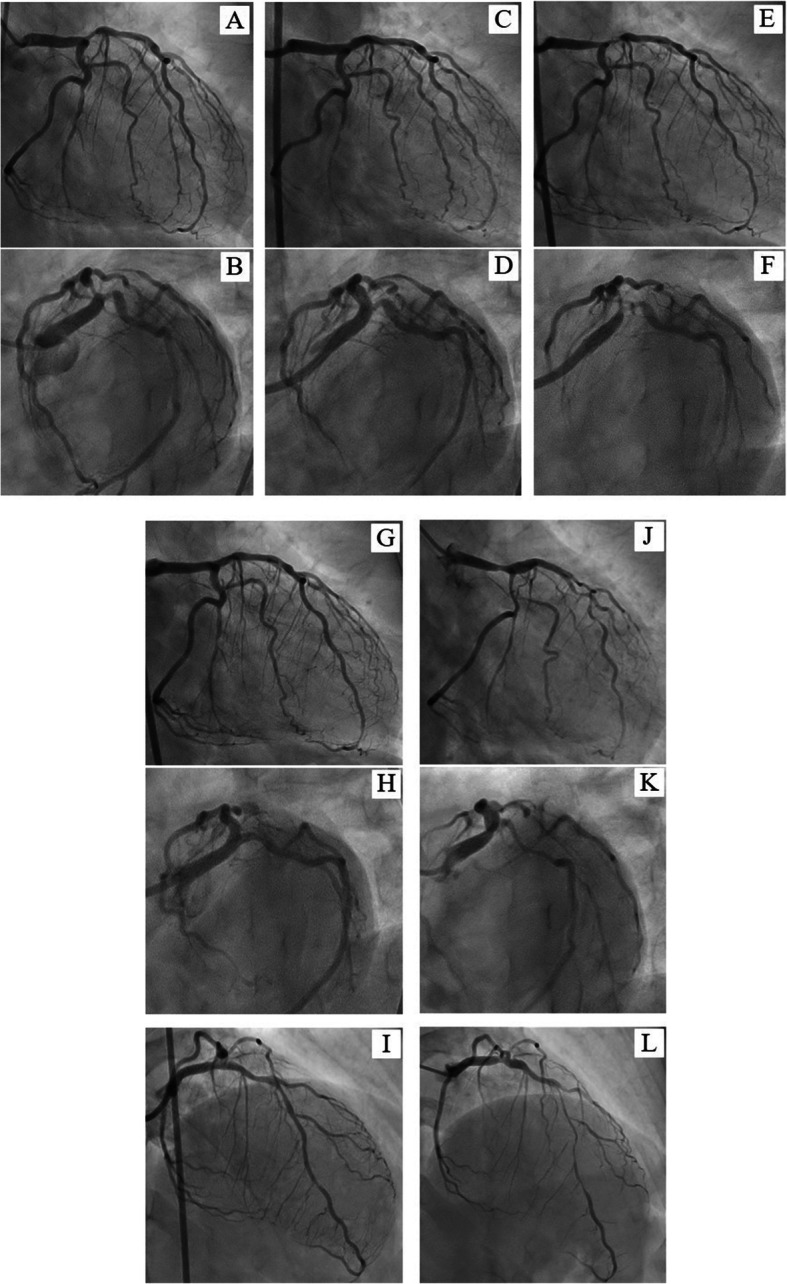
Coronary angiography. Initial angiography showed a focal stenosis of ostial LAD and a diffuse stenosis of high lateral branch of LCx (**a**, **b**). After the first percutaneous coronary intervention (PCI) by directional coronary atherectomy and paclitaxel-coated balloon (**c**, **d**). Five months later, a restenosis of the ostial LAD and progressed stenosis of LMCA and ostial LCx emerged (**e**, **f**). After the second PCI by directional coronary atherectomy and paclitaxel-coated balloon (**g**, **h**, **i**). At six months after the second PCI, restenosis of distal LMCA and ostial LCx, and diffuse stenotic progressions of the mid LAD and LCx were observed (**j**, **k**, **l**); coronary artery bypass graft was therefore performed. LAD: left ascending coronary artery, LCx: left circumflex coronary artery, LMCA: left main coronary artery, PCI: percutaneous coronary intervention

A 55-year-old woman was referred to our hospital because of chest pain on effort that had worsened in the last 3 months. The patient had no significant past medical history and was not on any medication. Family history revealed that her mother was diagnosed as having stable angina pectoris at 70. The patient had no evident physical findings of fever, lymphadenopathy, joint pain or left-right deference of blood pressure. Electrocardiogram showed regular sinus rhythm and no significant abnormalities (Fig. 1). The echocardiogram revealed normal left ventricular wall motion and no significant structural heart disease. Master’s double two-step test revealed ischemic ST changes, and CT coronary angiography showed a severe stenotic lesion at the ostial left anterior descending coronary artery (LAD). Laboratory examinations revealed a high level of serum low-density lipoprotein cholesterol (202 mg/dl), slightly elevated troponin I (68.2 pg/ml) and erythrocyte sedimentation rates (23 mm/1 h), and normal C-reactive protein level (0.21 mg/dl). The patient’s Achilles tendon was mildly thickened (9.3 mm) which led to a diagnosis of heterozygous familial hypercholesterolemia. Catheter coronary angiography showed a 90% stenosis of the ostial LAD and a 99% diffuse stenosis of the high lateral branch of the left circumflex (LCx) (Fig. 2a and b). The right coronary artery was hypoplasty and no stenotic lesions were observed. A diagnosis of effort angina pectoris caused by the stenotic lesion of the ostial LAD with heterozygous familial hypercholesterolemia was made at this time.

**Fig. 3:**
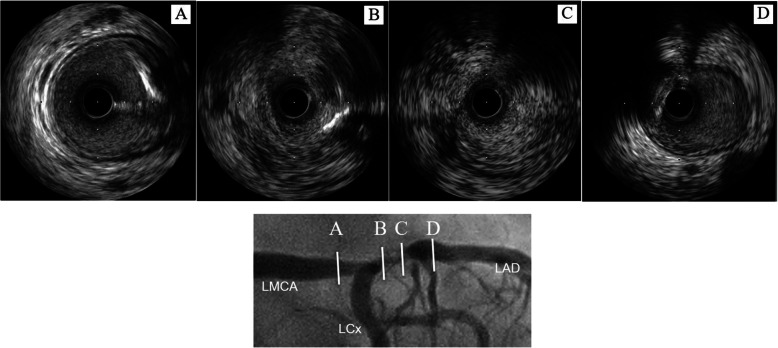
Intravascular ultrasound images. Intravascular ultrasound images of the stenotic lesion obtained during the first coronary intervention showed a fibrotic intimal thickness and an attenuated plaque was not seen. It is noteworthy that the media was partly unrecognizable and the echo intensity similar to fibrotic intimal thickening traversed from the intima to the adventitia, thereby causing the whole image of the coronary artery to become unclear

**Fig. 4:**
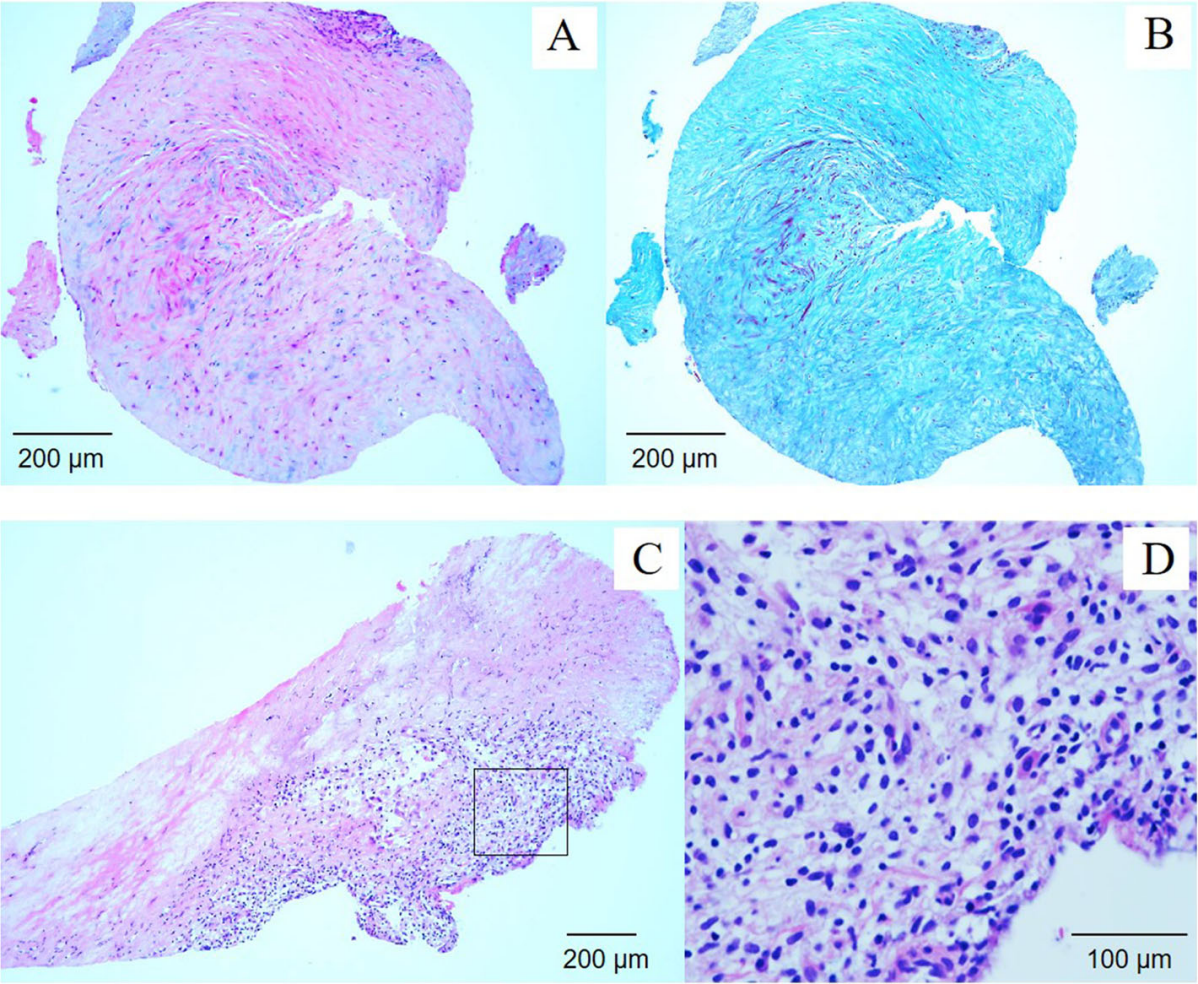
Histopathologic findings of the lesion obtained by the directional coronary atherectomy. Although the histopathologic findings of almost all specimens that retrieved by directional coronary atherectomy at the first coronary intervention revealed an intense fibrous intimal thickening without atheroma (**a**: Hematoxylin and eosin stain, **b**: Elastica masson stain), an accumulation of inflammatory cells with granuloma was observed in a small section of specimens obtained at the second directional coronary atherectomy (**c**: Hematoxylin and eosin stain, **d**: High-power field)

Percutaneous coronary intervention with coronary plaque atherectomy using directional coronary atherectomy (DCA) catheter (ATHEROCUT™, Nipro Corporation, Japan) followed by paclitaxel-coated balloon (SeQuent® Please, B. Braun, Melsungen, Germany) was performed on the LAD lesion, and plain old balloon angioplasty was performed on the high lateral branch lesion (Fig. 2c and d). The images obtained by 40-MHz IVUS catheter (Opticross®, Boston Scientific, Natick, MA) showed a fibrotic intimal thickening at the proximal LAD, and no attenuated plaque was observed. In addition, the following was observed at the ostial LAD; media, the echolucent band surrounding an intimal layer and an internal elastic lamina, was partly unrecognizable and the echo intensity similar to fibrotic intimal thickening traversed from the intima to the adventitia, thereby causing the whole image of the coronary artery to become unclear (Fig. 3). Histopathological study of the specimens retrieved by DCA revealed intense fibrous intimal thickening, and no atheroma was observed (Fig. 4a and b). Rosuvastatin and ezetimibe were administered, resulting in a decrease in serum low-density lipoprotein cholesterol level to 66 mg/dl (Table 1).

**Table 1:** Laboratory findings of cholesterol levels

	LDL-cholesterol (mg/dl)	HDL-cholesterol (mg/dl)
Before treatment	202	76
+ rosuvastatin	90	74
+ ezetimibe	66	76
+ evolocumab	11	64

*LDL* Low-density lipoprotein; *HDL* High-density lipoprotein

Legend: Low-density lipoprotein cholesterol level decreased from 202 mg/dl at baseline to 66 mg/dl after administration of rosuvastatin and ezetimibe. The intensive lipid lowering therapy with evolocumab, proprotein convertase subtilicin-kexin type 9 inhibitor, decreased further to 11 mg/dl

**Fig. 5:**
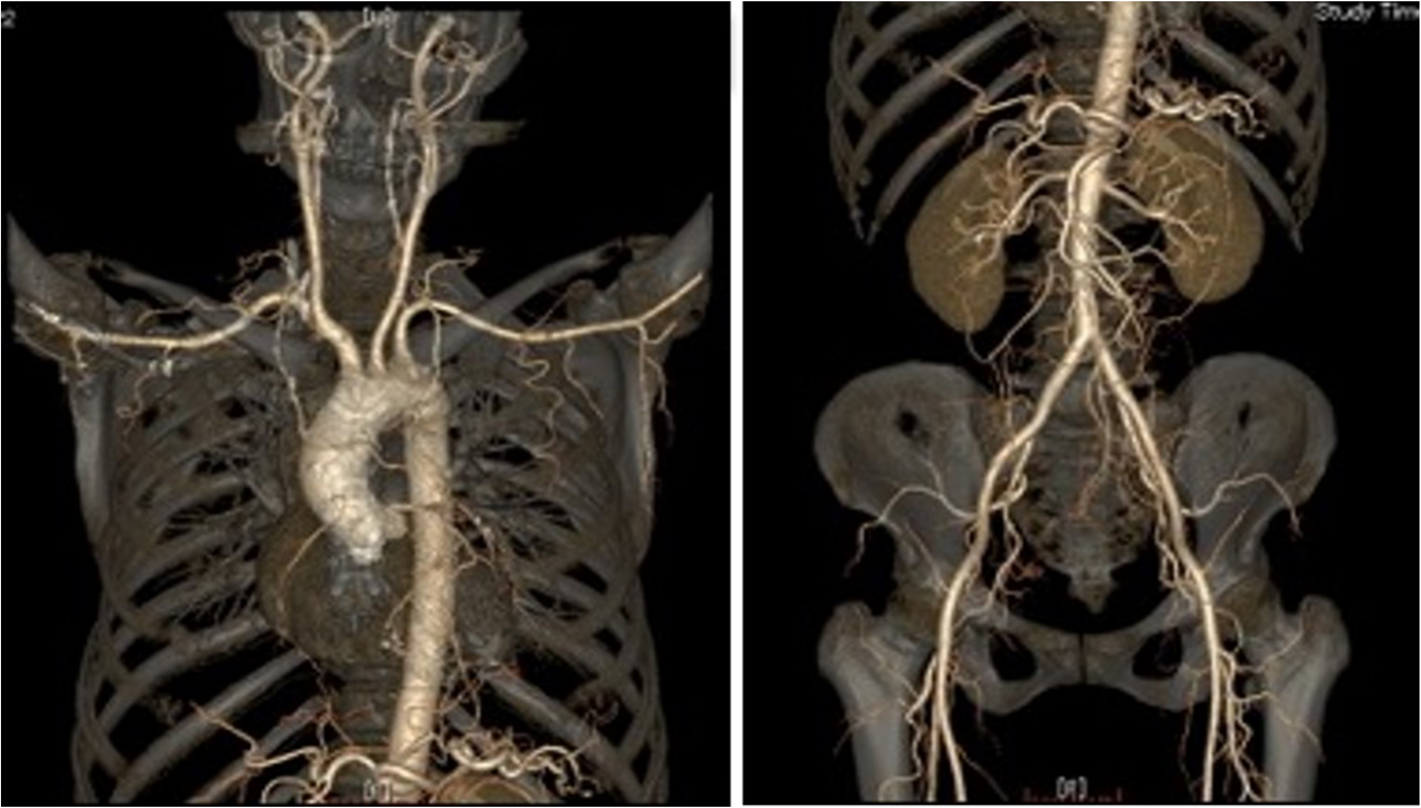
CT angiography. Systemic CT angiography revealed no stenotic or aneurysmal changes of arteries except for coronary arteries

Five months later, she suffered from chest pain on effort again and coronary angiography showed a restenosis of the ostial LAD, as well as a stenosis of the distal left main coronary artery (LMCA) and the ostial LCx (Fig. 2e and f). In IVUS findings, the media and the whole coronary appearance became unclear in more extensive range. Percutaneous coronary intervention with DCA followed by paclitaxel-coated balloon was done again from LMCA to ostial lesions of both the LAD and the LCx (Fig. 2g, h and i). Lipid-lowering therapy was intensified that evolocumab, proprotein convertase subtilicin-kexin type 9 inhibitor, decreased the low-density lipoprotein cholesterol level to 11 mg/dl (Table 1). Although the histopathological findings of the specimens retrieved via DCA revealed intimal fibromascular hyperplasia similar to that found by the first coronary intervention, an accumulation of inflammatory cells with granulomas was observed in a small section of specimens (Fig. 4c and d). Whole body CT angiography was performed to examine systemic arteritis, in which no stenotic or aneurysmal changes of the arteries were observed, except for the coronary arteries (Fig. 5).

**Fig. 6:**
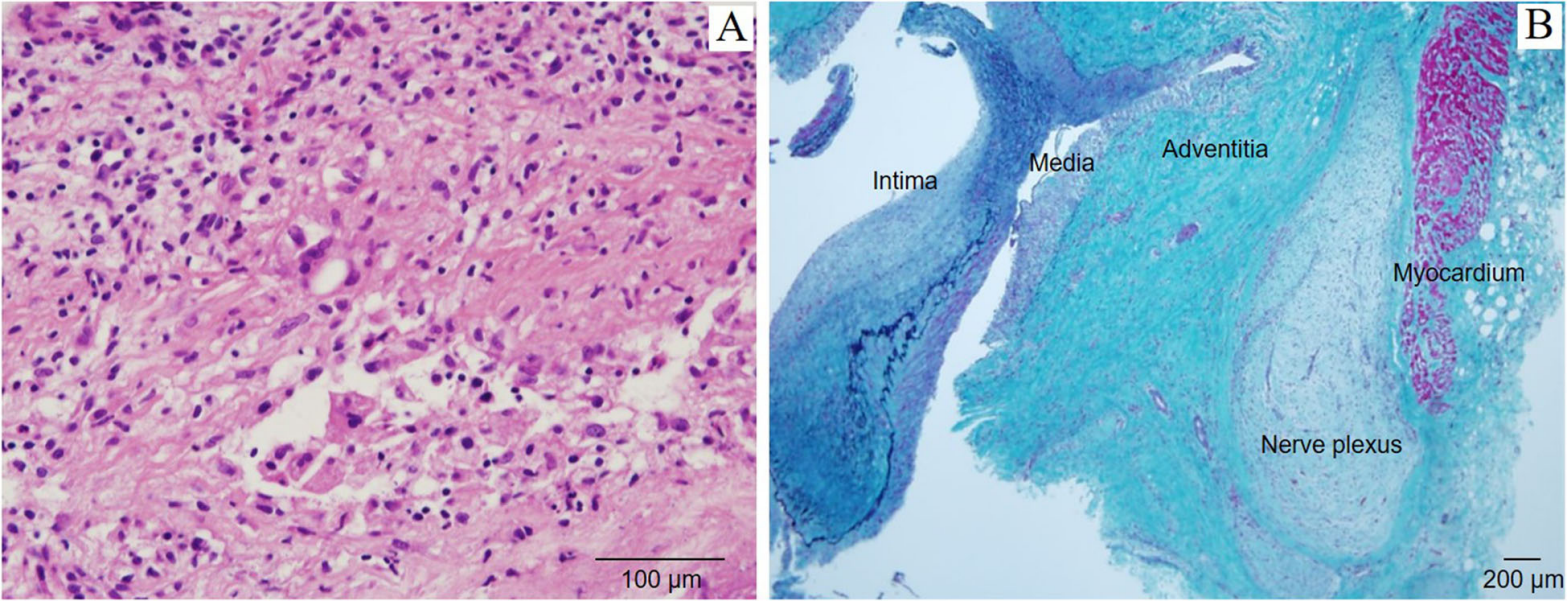
Histopathologic findings of the lesion obtained by the coronary artery bypass graft. Histopathlogical findings of specimens that was obtained during coronary artery bypass graft showed fibromuscular intimal thickness, granulomatous inflammation with giant cells at media (**a**). Extremely thickened adventitia with fibrosis involving the myocardium and the epicardial nerve plexus was also observed (**b**)

Single photon emission computed tomography myocardial perfusion imaging at 6 months after the second coronary intervention revealed significant apical and lateral wall ischemia. Coronary angiography showed the restenosis of the ostial LCx and the diffuse stenotic progressions of the mid LAD and LCx (Fig. 2j, k and l). Coronary artery bypass graft was then performed, in which a specimen of resected coronary artery was obtained from a stenotic lesion of a diagonal branch which included all three vessel layers. Histopathological examination showed fibromuscular intimal thickening and granulomatous inflammation with giant cells in the media; therefore, the external elastic band was partially destructed. Extremely thickened adventitia with fibrosis involving the myocardium and the epicardial nerve plexus was also observed (Fig. 6). These microscopic findings led to the diagnosis of isolated coronary Takayasu arteritis.

Systemic corticosteroid therapy (prednisolone 1 mg/kg/day) was then started. Since the administration of corticosteroids, at the time of writing, the patient has been free from chest pain for 1 year.

## Discussion

Takayasu arteritis is an idiopathic chronic inflammatory arteritis characterized by stenosis, occlusion or dilatation of the aorta and its major branches, and is predominantly prevalent among young women [1]. Coronary artery involvement has been reported in approximately 10 to 45% of Takayasu arteritis autopsy cases [2, 3], while over 70% of the coronary lesions were located at the coronary ostium [2, 4]. To make an accurate diagnosis of Takayasu arteritis, in addition to both clinical symptoms caused by the ischemia of particular organs and diagnostic vascular images (ultrasonography, CT angiography, magnetic resonance angiography or catheter angiography), several conditions that include atherosclerosis should be ruled out [5]. In the present case, there were no signs of Takayasu arteritis except for effort angina. Furthermore, the primary lesion characteristics that were isolated and non aorto-ostial lesion with hypercholesterolemia seemed to be effort angina with conventional atherosclerotic plaque, thus percutaneous coronary angioplasty and lipid lowering therapy were performed as usual. However, regardless of a remarkable mid-term result of a combination therapy of DCA followed by paclitaxel-coated balloon for distal LMCA bifurcated lesions [6], and the effectiveness of intensive lipid lowering therapy for reducing adverse cardiovascular events [7], the lesions in the present case showed restenosis and diffuse progression. The diagnosis of Takayasu arteritis was not made before histopathologic examination of resected coronary specimens that were obtained during coronary artery bypass graft.

**Fig. 7:**
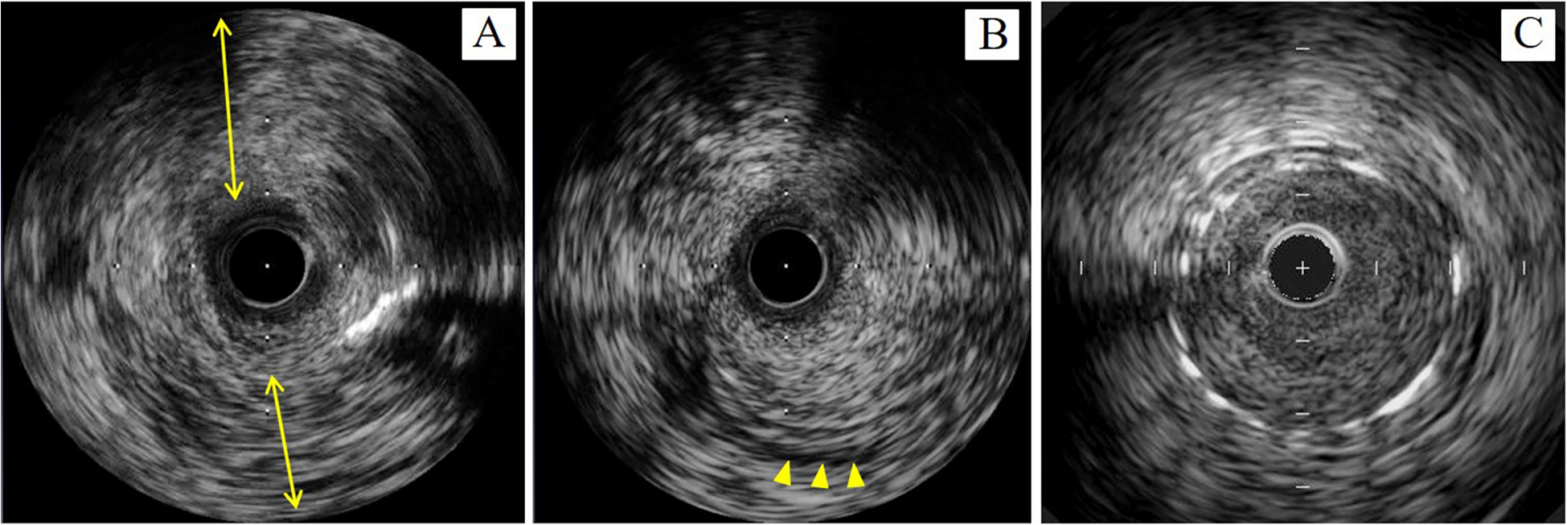
Comparison of IVUS images between Takayasu arteritis and typical neointimal hyperplasia observed in in-stent restenosis. In Takayasu arteritis, heterogeneous and relatively high echo were observed, and the most distinctive features are unrecognized media (arrowheads) and the expanse of fibrotic thickening from the media to the adventitia (arrows) (**a** and **b**). The typical findings in neointimal hyperplasia reveal homogeneous, concentric and echolucent patterns (**c**)

IVUS imaging of the lesion revealed intimal fibrotic thickening without lipid-rich plaque, in which the media was partly unrecognizable and the echo intensity similar to fibrotic intimal thickening traversed from the intima to the adventitia. The histopathologic characteristics of Takayasu arteritis are granulomatous inflammation of the media and adventitia with giant cells, diffuse productive inflammation consisting of lymphocytes and plasma cells infiltration, diffuse or nodular fibrosis accompanied by disintegration and loss of elastic fibers, and reactive intimal fibrotic thickening. These conditions caused marked intimal and adventitial fibrosis [8]. The disintegration and fibrosis of the media resulting in fibrosis of all three vessel layers may have led to characteristic IVUS findings. The intimal fibrotic thickening is commonly seen in neointimal hyperplasia at in-stent restenosis. The typical IVUS findings in neointimal hyperplasia are homogeneous, concentric and echolucent patterns [9]. On the other hand, IVUS findings of intimal thickening in Takayasu arteritis in the current case showed heterogeneous and a relatively high echo, and the most distinctive features are unrecognized media and the expanse of fibrotic thickening from the media to the adventitia (Fig. 7). Although the usefulness of ultrasonography for evaluating carotid vasculitis in Takayasu arteritis has been reported previously [10], to our knowledge, the present case report is the first to show IVUS images of coronary lesions in a case of Takayasu arteritis.

Directional coronary atherectomy is a useful method especially for bifurcated lesions [11]. The combination of DCA and paclitaxel-coated balloon may avoid complex stenting to LMCA bifurcation with an equivalent efficacy compared with a drug eluting stent [6]. Therefore, DCA with paclitaxel-coated balloon was used for the LMCA bifurcated lesion in the present case. Directional coronary atherectomy also plays a role in obtaining specimens of the coronary plaque for histopathologic examination. In the present case, though the specimens obtained at the first coronary intervention merely showed the fibrous intimal thickening, the specimens retrieved at the second intervention revealed an accumulation of inflammatory cells with granulomas in a small section. A previous study reported the usefulness of 18F-fluorodeoxyglucose-positron emission tomography for diagnosing coronary Takayasu arteritis [12], which possibly could have helped diagnosis before coronary bypass surgery in the present case.

Both Takayasu arteritis and familial hypercholesterolemia provoke arterial stenosis as a result of inflammatory disorder of the arterial wall; however, the correlation of these diseases is unclear [13, 14]. The pathologic backgrounds of these two diseases are different; lipid accumulation in vessel wall activates macrophages and yields the formation of the necrotic core in the state of hypercholesterolemia [15], whereas granulomatous inflammation of the media and adventitia with giant cells induces fibrotic thickening of the vessel wall in Takayasu arteritis. Considering these pathological differences and the finding that the intensive lipid lowering therapy could not inhibit the progression of arterial inflammation in the present case, there may be little relationship between familial hypercholesterolemia and Takayasu arteritis.

## Conclusion

We here described an extremely rare case of isolated and non aorto-ostial coronary Takayasu arteritis, and IVUS images of coronary lesions in Takayasu arteritis. Characteristic IVUS images may help for diagnosing coronary arteritis.

## Data Availability

Data are available from Takeshi Shimizu (e-mail: takeshis@fmu.ac.jp) upon reasonable request and with permission of Hoshi General Hospital.

## Abbreviations

LAD: Left anterior descending coronary artery
LCx: Left circumflex coronary artery
CT: Computed tomography
IVUS: Intravascular ultrasound
DCA: Directional coronary atherectomy
LMCA: Left main coronary artery
